# Multimodal Treatment of Neck Pain in Multiple System Atrophy

**DOI:** 10.1002/mdc3.70497

**Published:** 2026-01-05

**Authors:** Bianca Caliò, Svenja Schmidt, Alessandra Fanciulli

**Affiliations:** ^1^ Department of Neurology Medical University of Innsbruck Innsbruck Austria

**Keywords:** antecollis, multiple system atrophy, orthostatic hypotension, physiotherapy

Pain is a distressing symptom affecting up to 87% of individuals living with multiple system atrophy (MSA).^1^ Despite its substantial impact on quality of life, pain remains frequently underrecognized and inadequately treated in MSA.^1^

Recent studies showed that pain most frequently affects the neck and shoulder region (58%) in MSA, with both motor and non‐motor contributing factors.^1^, ^2^ Among the non‐motor features, orthostatic hypotension (OH) may present as *coat hanger pain*, an unpleasant sensation in the neck and shoulders caused by muscle hypoperfusion in the upright position, which typically improves when sitting or lying down.^3^ *Antecollis*, an abnormal forward flexion of the neck, is a postural deformity highly suggestive of MSA when developing in individuals with early parkinsonism.^4^ It is dystonic in most cases and represents an important cause of pain, contributing to the overall disease burden.^4^

Pain treatment options are heterogeneous and often not tailored to the specific needs of MSA individuals. Here we present a multimodal approach to neck pain developed *for* and *in collaboration* with participants to the MeDeMSA Care study.^5^ This 18‐month, monocentric, open‐label study evaluates the impact of personalized multidisciplinary care, with integrated palliative and telemedicine support, on the quality of life of MSA individuals. Monthly online consultations with neurologists, physiotherapists, and other healthcare providers facilitate engagement with the study participants in their home environment. This closer and more confidential interaction enabled the study team to learn about strategies that resilient individuals with MSA and their families had developed to cope with neck pain.

## Case Report

A 63‐year‐old woman with a four‐year history of parkinsonian‐type MSA (MSA‐P) reported severe neck pain associated with pronounced antecollis, which also impaired eating and communication. She additionally experienced OH with coat‐hanger pain upon standing. Following the MeDeMSA operational protocol,^5^ OH was treated with a combination of non‐pharmacological and pharmacological measures (i.e., etilefrine). For antecollis, we optimized the dopaminergic regimen and recommended physiotherapy, along with occupational and speech therapy exercises. During one of her telemedicine visits, she proudly showed to the team her husband's ingenious solution to alleviate antecollis: a backpack frame fitted with a cooking spoon anchored to it and secured to the back of her head with a bandana.

**Figure 1:**
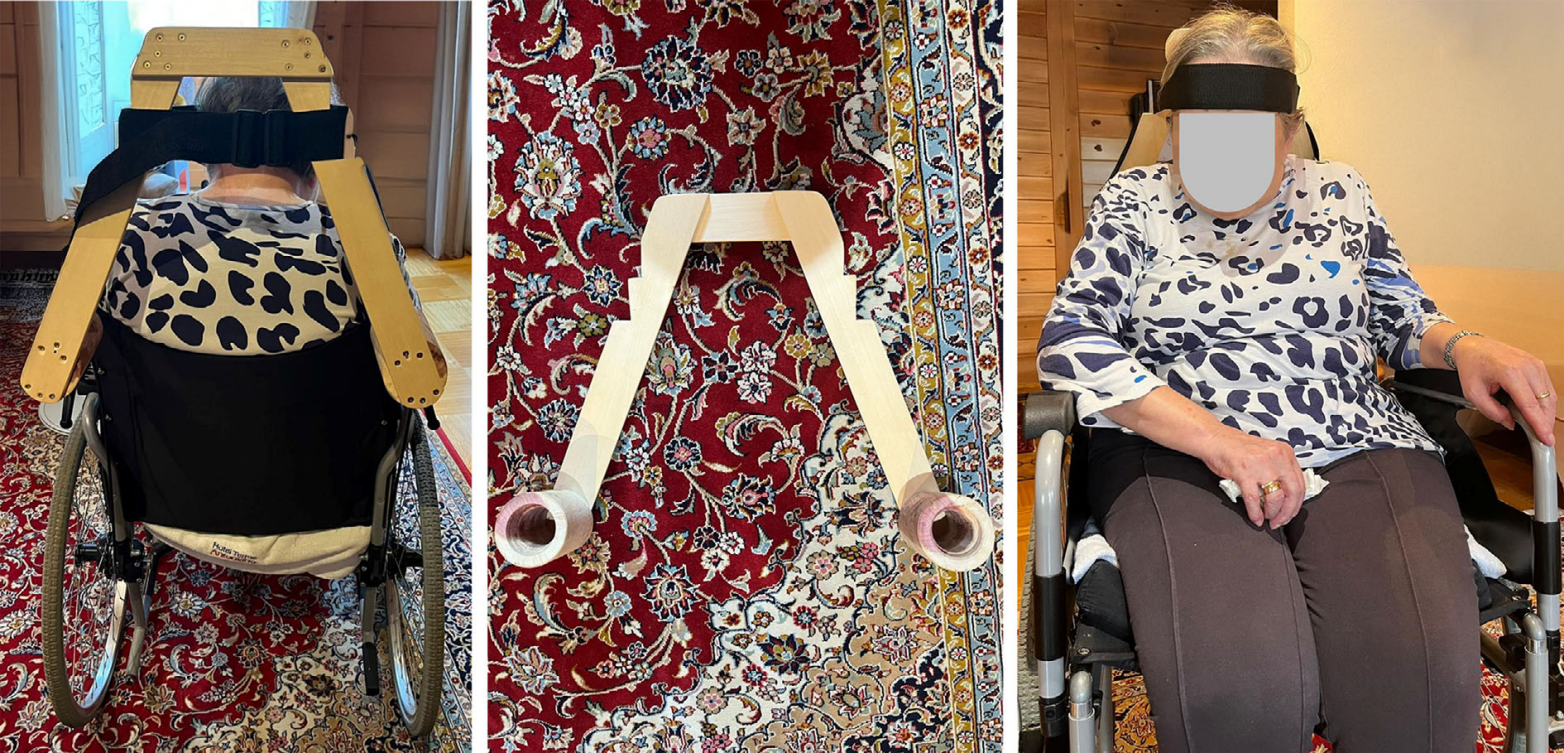
Wooden‐made orthosis with elastic band.

Hers was not the only case of caregiver‐initiated strategies to mechanically counteract the neck flexion. During another telemedicine visit, we learned that a retired carpenter, whose wife was living with advanced MSA, had carved a wooden support attached to the wheelchair handles and connected to her head with an elastic band (Fig. 1).

Building on these patient and caregiver insights, the MeDeMSA team physiotherapists searched for commercially available solutions. After careful testing, our MSA‐P patient was provided with an orthosis that allowed both horizontal and vertical head movements, yet preventing neck drop toward the thorax^6^ (see Video 1). This intervention effectively reduced her neck pain and reportedly improved both communication and nutrition.

**Video 1:** Commercially available orthosis for antecollis support. The orthosis consists of a torso support (Spinomed®, Medi GmbH), a screwed joint holder with integrated quench joint (DYNA 35, Caroli GmbH), and a head connection. The orthosis allows an anteroflexion and lateral rotation of the head, while preventing excessive cervical flexion toward the thorax.

## Discussion

A community‐based survey showed that individuals with MSA often manage their pain alone, primarily using non‐steroidal anti‐inflammatory drugs and opioids.^1^ In the case of antecollis‐ or OH‐related neck pain, opioids are not only barely effective, but may even worsen symptoms, due to their hypotensive effect and associated weakness.^7^ Because coat‐hanger pain is uncommon in the general population, patients may not recognize its association with hypotensive triggers, such as standing, heat, meals, alcohol or physical exertion. New or increased neck pain should therefore always prompt a re‐evaluation of OH severity in MSA individuals. If an association with hypotension is found, non‐pharmacological and pharmacological measures to stabilize blood pressure should be prioritized to target the underlying cause and avoid inappropriate analgesic intake.^8^

Concerning antecollis, a dual effect of dopaminergic drugs may be observed: in some cases, particularly at early MSA stages, they may provide benefit, but they may frequently exacerbate craniocervical dystonia in others, warranting a regular reassessment of L‐Dopa effect.^9^ Physiotherapy can additionally mitigate symptoms through targeted neck muscle training, including extensor strengthening, flexor stretching, and extension mobility exercises.^10^

Beyond pain, antecollis impacts eating, eye contact, breathing, simple daily chores like picking a book from a shelf, and increases the risk of falls. This combination of physical discomfort and functional limitation adds considerable psychological distress. In this context, multidisciplinary care teams facilitate tailored supportive interventions, including orthoses that provide adequate head support while maintaining freedom of movement.

Effective management of complex, evolving symptoms like pain, requires continuity of care and a good understanding of the patient's perspective. Due to time constraints, these aspects often remain unaddressed in outpatient settings. We learn from the ongoing MeDeMSA Care study that telemedicine helps to overcome these barriers by enabling more regular, bidirectional exchange between patients, caregivers and healthcare professionals. By bringing the individual home environment into view, telemedicine often uncovers adaptive strategies and unmet needs that remain otherwise unseen in clinical settings, ultimately facilitating the development of tailored technological and therapeutic solutions.

## Author Roles

(1) Research Project: A. Conception, B. Organization, C. Execution; (2) Manuscript Preparation: A. Writing of the First Draft, B. Review and Critique.

B.C.: 1B, 1C, 2A.

S.S.: 1B, 1C, 2B.

A.F.: 1A, 1B, 1C, 2B.

## Disclosures

**Ethical Compliance Statement:** The MeDeMSA Care Study protocol was approved by the Ethical Committee of the Medical University of Innsbruck (EK No.: 1225/2023). Written informed consent for participation in the study was obtained from all participants for the use of images and videos and appropriately documented. We confirm that we have read the Journal's position on issues involved in ethical publication and affirm that this work is consistent with those guidelines.

**Funding Sources and Conflict of Interest:** This academic study was funded in whole by the Austrian Science Fund (FWF, 10.55776/FG27 MeDeMSA Medical Decision Making in Multiple System Atrophy). For open access purposes, the author has applied a CC BY public copyright license to any author accepted manuscript version arising from this submission. The authors declare that there are no conflicts of interest relevant to this work.

**Financial Disclosures for the previous 12 months:** BC was supported by the Austrian Science Fund (FWF, FG 2700). SS has no disclosures to report. AF reports royalties from Springer Verlag, speaker fees and honoraria from Abbvie, Bial, CNSystems, Desitin, Elsevier, Ferrer, Ionis, Medtronic, Sanofi, Theravance Biopharma, Austrian Autonomic Society, International Parkinson Disease and Movement Disorders Society, and research grants from the European Reference Network for Rare Neurological Disorders, Medical University of Innsbruck, Mission MSA and Dr Johannes and Hertha Tuba Foundation, outside of the present work.

## Data Availability

The data that support the findings of this study are available from the corresponding author upon reasonable request.
